# Coverage and Quality of Antenatal Care Provided at Primary Health Care Facilities in the ‘Punjab’ Province of ‘Pakistan’

**DOI:** 10.1371/journal.pone.0113390

**Published:** 2014-11-19

**Authors:** Muhammad Ashraf Majrooh, Seema Hasnain, Javaid Akram, Arif Siddiqui, Zahid Ali Memon

**Affiliations:** 1 Allama Iqbal Medical College, Lahore, Pakistan; 2 Population Survives International – Greenstar, Karachi, Pakistan; The Hospital for Sick Children, Pakistan

## Abstract

**Background:**

Antenatal care is a very important component of maternal health services. It provides the opportunity to learn about risks associated with pregnancy and guides to plan the place of deliveries thereby preventing maternal and infant morbidity and mortality. In ‘Pakistan’ antenatal services to rural population are being provided through a network of primary health care facilities designated as 'Basic Health Units and Rural Health Centers. Pakistan is a developing country, consisting of four provinces and federally administered areas. Each province is administratively subdivided in to ‘Divisions’ and ‘Districts’. By population ‘Punjab’ is the largest province of Pakistan having 36 districts. This study was conducted to assess the coverage and quality antenatal care in the primary health care facilities in ‘Punjab’ province of ‘Pakistan’.

**Methods:**

Quantitative and Qualitative methods were used to collect data. Using multistage sampling technique nine out of thirty six districts were selected and 19 primary health care facilities of public sector (seventeen Basic Health Units and two Rural Health Centers were randomly selected from each district. Focus group discussions and in-depth interviews were conducted with clients, providers and health managers.

**Results:**

The overall enrollment for antenatal checkup was 55.9% and drop out was 32.9% in subsequent visits. The quality of services regarding assessment, treatment and counseling was extremely poor. The reasons for low coverage and quality were the distant location of facilities, deficiency of facility resources, indifferent attitude and non availability of the staff. Moreover, lack of client awareness about importance of antenatal care and self empowerment for decision making to seek care were also responsible for low coverage.

**Conclusion:**

The coverage and quality of the antenatal care services in ‘Punjab’ are extremely compromised. Only half of the expected pregnancies are enrolled and out of those 1/3 drop out in follow-up visits.

## Introduction

Low up take of Antenatal care (ANC) is an important determinant of high maternal mortality rate in developing countries and is one of the basic components of maternal care on which the life of mothers and babies depend [1]. According to WHO, 536000 women die every year in the world from causes relating to pregnancy, childbirth or postpartum. Ninety nine percent of these deaths occur in the developing countries. The majority of these deaths could be avoided if women had access to quality medical care during pregnancy, childbirth and postpartum [2]. ANC services help pregnant women by identifying complications associated with pregnancy or diseases that might adversely affect the pregnancy [3]. The ANC coverage in the Punjab province of Pakistan is only 53% and there isinequity in provision of ANC services to rural population as depicted by some studies which is 50% in comparison to 71% of urban women. About 25% of women in rural and urban areas of Punjab consulted health care providers at a public sector health care facility [4]. Multiple reasons have been reported for low coverage of ANC services that includes the weak health systems, inadequate training [5] and inaccessibility of health facilities and ANC services in Pakistan are widely cited as being responsible for a high proportion of maternal and infant deaths [6]. However, in recent decades certain low income countries have witnessed dramatic improvements in publically funded health care delivery system. For instance in Sri Lanka maternal health care is provided free of charge to all women and in 2000, 94.5% of expected mothers had visited an antenatal care clinic at least once [7]. Social developmental factors like education markedly influence the ANC coverage in low income countries for instance in a study in Ethiopia ANC utilization increases by skilled care providers as the level of education of woman increases. Only 25% of women who had no education used skilled ANC attendants compared with 90% of those who had higher than secondary level education [8]. Introduction of group ANC sessions at public health centers in Iran showed a significant improvement in patient satisfaction and mean patient satisfaction score increased for women participating in group sessions compared to individual care. Significant improvements were seen in number of women receiving full number of ANC visits. Further, increase in birth weight and use of supplements was observed among the ladies receiving group ANC sessions [9]. An intervention in India for improvement of ANC through ensuring staff had necessary equipment, increased monitoring and supervision, focused training, re-organisation of outreach clinics and scheduling Quality of care increased as more than 60% of women received all ANC components. Significant increase in blood pressure and urine testing increased significantly. Attendance at clinics also increased by 30% [10].

In a study of expectant mothers in an urban squatter settlement of Karachi, indicated that 49% received no antenatal care, even in the presence of no cost and low cost public health sector ANC services [11]. A cross-sectional community-based study in Sudan reported that out of 900 pregnant women, 811 (90%) had at least one visit. Only 11% of the women had ≥ four antenatal visits [12]. In another study conducted in North West of Pakistan about the utilization of prenatal care services by the rural women, it was reported that only 28.1% (34/121) pregnant women of Sarbund village utilized prenatal care service in either private (6/121; 0.5%) or public sectors 28/121; 23.1%) [13]. Pakistan Demographic and Health Survey confirms that maternal deaths are not merely a result of treatment failure; rather they are the final outcome of a complex interplay between a myraid of social, cultural and economic factors [14].

Client satisfaction is the litmus test that enables health programs to assess the impact of their service; hence it is an integral part of the quality assurance process of health delivery. In recent years, there has been an increasing demand of accountability and productivity by consumers. It is now a global trend in health care development towards integrating subjective user satisfaction into the evaluation of medical service quality [15]. Patients' voice must begin to play a greater role in the design of health care service delivery process in the developing countries [16]. The satisfaction of female clients of ANC has been studied in the past in other countries [17]. A study in India reported that the overall summative index for clinical quality using a scale of 0–5 was 2.1% in the North and 4.1 in the South [18]. Similarly in another studyin Oman the overall satisfaction for antenatal care was excellent in 49 (CI 48.5–69.6) respondents. Out of 89 women, 67 (81%) were satisfied with the services because of the attitude of the doctors and nurses. The main causes of dissatisfaction were the laboratory services and overcrowding in the morning hours [19]. However in a study conducted at a Hyderabad, Sindh, half of the women in study sample were satisfied with the overall care provided to them. The routine antenatal investigations were provided to majority of women like urine, blood, antenatal examination and blood pressure. About 86.2% women said that they have to wait for more than two hours for checkups. Regarding satisfaction with getting medicine 63% were found dissatisfied, 75% of women did not have complete tetanus vaccine. Only 31% received instructions about perinatal care, 46% received information about exercise and 36% were reassured about discussing fear and anxiety [20].

In ‘Punjab’ province of Pakistan, there is network of PHC health facilities named as Rural Health Centers (RHCs) and Basic Health Units (BHUs). RHCs are relatively larger PHC facilities serving 10–50 thousands rural population while BHUs are relatively smaller health facilities providing PHC services to 5000 to 10,000 rural populations. One BHU is available up to almost each ‘Union Council’ level. There is uniform package of health services, infrastructure, physical and human resources. Medical Officers are in-charge of RHCs/BHUs and ANC services are supposed to be provided by the Lady Health Visitors (LHVs) at these facilities. This study is an attempt to evaluate the coverage and quality of antenatal care being provided to the rural population of this province. Although the study was focused on antenatal care but it will reflect the overall gaps in the health system of Pakistan to be bridged through appropriate interventions. The objectives of the study were:

1. To assess the proportion of expected pregnancies enrolled for ANC services in the catchment areas of PHC health facilities.
2. To assess the proportion of the enrolled clients coming for follow-up ANC visits.
3. To assess the quality ANC services delivery process in terms of assessment, treatment and counseling of the clients.
4. To explore and identify the causes of low coverage and quality of services at managerial, provider and client level.

## Methods

A cross-sectional study was conducted to assess the coverage and quality of ANC services in first-level health care facilities. Both quantitative and qualitative research methods were used to undertake this study. The study proposal was approved by National Bioethics Committee (NBC) Pakistan vide Ref No.4-87/09/NBC-36/RDC/7451 and the inception of the study was undertaken after the approval. A multi-stage sampling procedure was adopted to select the districts and primary level health facilities from each district. In first stage all the districts of Punjab were ranked from 1 to 36 on the basis of a composite indicator which was developed from eight socio-demographic indicators [21]. Total nine out of thirty six districts were selected from the Punjab province so that three district from each high, medium and low social stratum could be included in the study. In the second stage of sampling, 19 health facilities (17 BHUs and 2 RHCs) were randomly selected from each district. Initially a total 171 health facilities BHUs/RHCs were accessed for completion of facility performance data. Approval for health facility assessment and interviews for health care providers was taken from Punjab Health Department. A consent form was developed in the local language that was approved by National Bioethics Committee (NBC) Pakistan. Informed verbal consent was taken from the clients and health care providers for interviews. The Consent form for each interview was filled and signed by a witness at health facilities and its record was maintained along with other data forms. Written consent was not possible for the reason that majority of the clients were illiterate (unable to read or write). During the survey in the first attempt, data collection from 151 health care facilities was completed and an additional 20 alternate health facilities were visited by teams in second attempt due to non-availability of providers or clients to complete the target sample. Client exit interviews were conducted after each client-provider interaction to assess the client satisfaction. The client exit interviews included the questions about the behavior of provider, ANC examination, time spent and the overall perception of satisfaction for the services provided.

Focal Group Discussions (FGDs) and in-depth interviews were conducted in qualitative part of assessment. Nine FGDs for clients and nine for providers were conducted in each of the nine sampled districts. The criteria for the selection of the clients was pregnant women who had already experienced at least one pregnancy and birth process, belonged to lower or middle social class and these clients came from the catchment area of health facility. There were 12 clients in each FGD session who were identified by the 'Lady Health Workers (LHW) in the catchment areas of the health facilities. LHWs enroll the pregnant ladies keep the records and refer them to the BHUs and RHCs for ANC services. The selection criterion for health care providers (Lady Health Visitor) was that their duration of working in these facilities was more than six months. The participation of health care providers was voluntary and the invitations were extended to them with permission of management of provincial health department. A total of 12 LHVs were included in each FGD. One FGD was conducted for Medical Officers/Women Medical Officers at the provincial level. In-depth interviews were conducted with district health managers (Executive District Officers for Health/District Officers for Health) and the provincial managers (Director General Health, Director MNCH Program and Director MIS Cell.

The data was collected on objectively developed semi-structured questionnaire from October-November 2010. Total eight teams were engaged for data collection and each team consisted of one team leader/supervisor and two surveyors/interviewers. A qualitative assessment team comprised of two sociologists and one public health consultant. All teams were provided extensive training in two separate 3-day workshops, one for qualitative and other for quantitative assessment teams. Field monitoring was carried out by two Regional Coordinators, a Public Health Consultant and the Principal investigator. Qualitative data was collected from December 2010 to February 2011.

Univariate analysis was done to describe the frequency and percentage by type of facility and by districts. ANC coverage was evaluated by examining the record of services provided to ANC clients at health facilities. For this purpose, annual ANC first enrolment and ANC follow-up visits were used as indictors. The percentage of the annual expected [22] pregnancies in the catchment area of health facility who reported first time for ANC services during the year before survey (2010) was operationally defined as ANC Enrollment. Percentage of the annual expected pregnancies in the catchment area of health facility reported for follow-up ANC services during the year before survey (2010) was defined as ANC-follow-up visits [19]. It was not possible to calculate the number of visits of a particular client from this data source. The revisits reported from this column are also termed as revisits in the reporting tools of Punjab MIS. The quality of ANC services was based on the steps followed by the health care-providers. A standard check list of steps to be followed health care provider, was developed by consultation of WHO criteria and Minimum Service Delivery Standards (MSDS) adopted by Maternal and Newborn and Child Health Program (MNCH) of the Punjab. This check list was used to observe the client provider interaction during ANC service provision. The providers that follow more than 80% of the steps enlisted in the checklist of assessment, treatment and counseling of the clients were ranked as good, between 60–80% as average and less than 60% as poor to measure the quality of ANC.

The qualitative data processing was initiated in the field with development of field transcripts by the interviewers/facilitator. A qualitative analysis framework was prepared for each target group e.g. clients, providers, facility in-charges, district health managers and provincial stakeholders. The findings were categorized under the main titles of the themes included in qualitative tools relevant to specific participants in FGDs. Findings were analyzed by inductive reasoning approach and qualitative assessment report was compiled under the supervision of a senior sociologist by consulting the individual session reports, transcripts/notes of FGDs and transcripts/notes of all in-depth interviews. Broadly, the analysis of data was arranged by the objectives of the study as stipulated in the research proposal.

## Results

### ANC coverage

**Figure 1:**
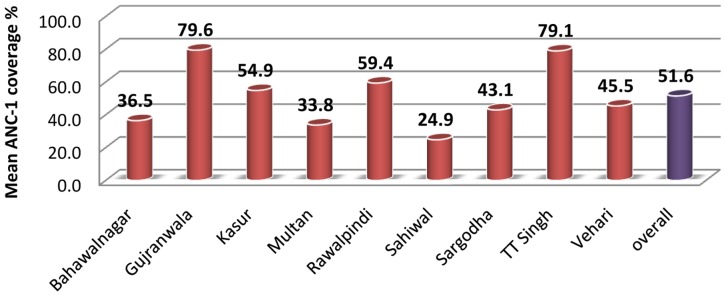
District-wise and overall mean annual ANC-1 coverage in PHC health facilities.

**Figure 2:**
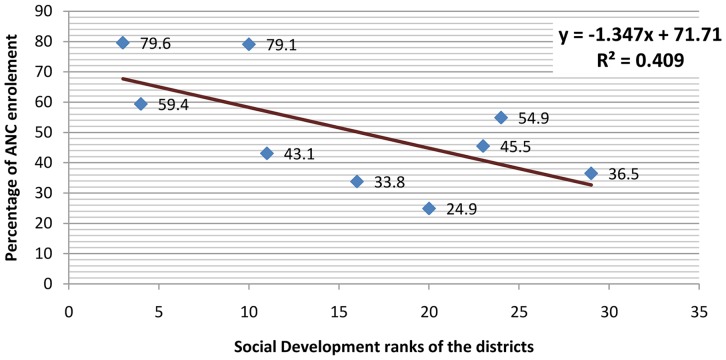
Association of Social Development Ranking with the District ANC coverage.

The overall enrollment was 51.6% in sampled districts of the Punjab province including the 17 redundant health facilities where no services being provided. Wide range of variations was observed among districts in ANC enrollment. Maximum coverage was observed in district Gujranwala at 79.6% followed by district Toba Tek Singh with 79.1% ANC-1 coverage annually. Minimum coverage was reported from district Sahiwal as 24.9% followed by district Multan as 33.8%. Out of the whole sample, five districts – Multan, Vehari, Bahawalnagar, Sahiwal and Sargodha – had coverage below overall mean and four districts – Gujranwala, Rawalpindi, Toba Tek Singh and Kasur – had ANC coverage above overall mean. (Fig. 1) The regression analysis indicates that there was 1.347% decrease in ANC coverage with one digit rise in ranking of the districts with constant value at base line of 71.7%. The Pearson Product Correlation coefficient, r = 0.64 and r2 = 0.41. All the districts in the Punjab were ranked from 1 to 36 according to a composite indicator based on adult literacy, primary school enrolment, under-five mortality, prevalence of under-nutrition, adequate water and sanitation, the percentage of deliveries with a skilled birth attendant, and use of contraceptive. The rank 1 mean most developed district and rank 36 mean least developed district. The higher ranks number are indicating lower social developmental index and low coverage. In other words with one degree increase rank mean decrease in social development and there is decrease in 1.347% ANC coverage and 71.7% is the highest base line coverage on the line of best fit of regression line. (Fig. 2).

ANC-enrollment percentage was increased from 51.6% to 55.9% when the data from 17 alternative non-redundant facilities was added to the analysis. The overall dropout was 32.8% in subsequent visits after the ANC visit in the health facilities. The drop was observed in all the districts except ‘Rawalpindi’ where there was a 29.1% increase in the number of follow-up visits. The reason for increase follow-up visits in this district was the availability of WMOs, free ultrasound services and accessible locations of some of the RHC health facilities.

Health facilities were ranked according to monthly percentage of expected pregnancies enrolled for ANC in the catchment area of health facilities. Health facilities enrolling more than 80% of expected pregnancies were ranked as good, 60–80% as average and less than 60% as poor. ANC coverage facility ranking revealed that overall only 24% facilities achieved a good ranking, 13.5% had average and 62.6% facilities had poor ranking in monthly ANC-1 coverage. ANC-follow-up/revisits followed a similar trend. (See Table 1).

**Table 1:** Ranking of health facility by ANC-enrolment and follow-up visits in the catchment areas.

	Overall		RHCs		BHUs	
Number of facilities	171		18		153	
	n	%	n	%	n	%
**Ranking of facilities by monthly ANC enrolment**						
Good coverage >80%	41	24	10	56	31	20
Average coverage ≥60% ≤80%	23	13	3	17	20	13
Poor coverage <60	107	63	5	28	102	67
**Ranking of facilities by monthly ANC-follow-up/revisits**						
Good coverage >80%	27	16	6	33	21	14
Average coverage ≥60% ≤80%	19	11	3	17	16	10
Poor coverage <60	125	73	9	50	116	76

The inferential analysis shows that <80% coverage was observed in 76% of facilities with 95% confidence limits from 69% to 82%. In comparison by type of facility, 56% of RHCs were providing good coverage compared to only 20% of BHUs. Thus more than half of RHCs had a good ranking for coverage while 80% BHUs had poor or average ranking.

### Service quality

**Figure 3:**
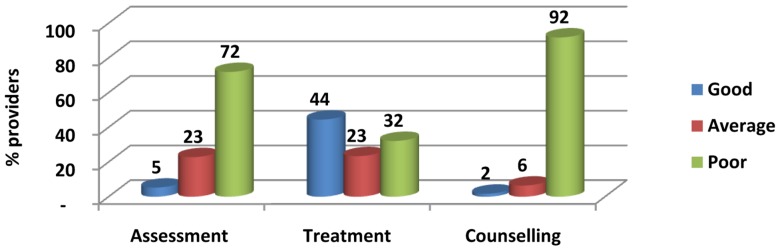
Ranking of ANC Services for Assessment Treatment and Counselling.

The appropriate history taking was followed by less 30% of providers in the context of asking about previous still births, bleeding, assisted deliveries, abortions, headache/blurring of vision, swelling and fever. Feeling of baby movement was enquired from only 9% of the clients. Most of the history steps were falling less than 50% range. The standard protocols for clinical examination were not being fulfilled in most of the cases. The breast examination was practiced in 10%, looked for edema 25%, test advised 21%, heard fetal heart sound in 51%, abdomen was palpated in 76% and BP was recorded in 74% of the services provided. The overall ranking of quality of clinical assessment was poor in 72%, average in 23% and good in only 5% of the services provided. (Fig. 3, Table 2).

**Table 2:** Quality of ANC services and client satisfaction (Overall and by types of facilities).

	Overall		RHCs		BHUs	
Number of facilities	171		18		153	
	n	%	n	%	n	%
**Areas of client assessment by history**						
Age of client	139	81	15	83	124	81
Use of medication by client	77	45	8	44	69	45
Date of LMP asked	103	60	10	56	93	61
Previous pregnancies	111	65	11	61	100	65
Previous still births/neonatal deaths	45	26	6	33	39	25
Heavy bleeding	23	13	5	28	18	12
Assisted deliveries	30	18	4	22	26	17
Abortions	42	25	5	28	37	24
Asked about bleeding	47	27	6	33	41	27
Fever	57	33	6	33	51	33
Headache/blurred vision	45	26	5	28	40	26
Swelling	59	35	6	33	53	35
Tiredness/breathlessness	56	33	6	33	50	33
Felt fetal movement	105	61	12	67	93	61
Mentioned bleeding	16	9	4	22	12	8
Mentioned fever	35	20	4	22	31	20
Mentioned headache/blurred vision	30	18	3	17	27	18
Mentioned swelling	36	21	5	28	31	20
Mentioned tiredness/breathlessness	52	30	6	33	46	30
Mentioned Fetal Movements	79	46	9	50	70	46
**Areas of client assessment by examination**						
Record BP	127	74	9	50	118	77
Recorded weight	102	60	7	39	95	62
Palpated abdomen	130	76	16	89	114	75
Foetal heart sounds heard	88	51	12	67	76	50
Looked for oedema	42	25	5	28	37	24
Breast exam done	17	10	3	17	14	9
Tests advised	36	21	9	50	27	18
ANC card filled	73	43	10	56	63	41
Talked about TT	109	64	11	61	98	64
**Areas of client counselling**						
Advice about food	108	63	10	56	98	64
Importance of TT vaccination told	48	28	6	33	42	27
Advised TT	97	57	7	39	90	59
Talked about Contraception	10	6	2	11	8	5
Talked about breast feeding	10	6	2	11	8	5
Place of delivery	53	31	7	39	46	30
Arrangement for transport	35	20	4	22	31	20
Who will accompany her	9	5	1	6	8	5
Time to reach place of delivery	9	5	1	6	8	5
Cost of delivery	11	6	1	6	10	7
Give next visit date	136	80	12	67	124	81
**Ranking of client counselling**						
Adequate counselling practices >80%	3	2	1	6	2	1
Average counselling practices ≥60% ≤80%	11	6	2	11	9	6
Poor counselling practices <60%	157	92	15	83	142	93
**Areas of client treatment**						
Iron pills prescribed	118	69	12	67	106	69
Folic acid tablets prescribed	109	64	10	56	99	65
TT injection prescribed	106	62	11	61	95	62
Anti-malarial prescribed	4	2		–	4	3
**Ranking of client treatment**						
Good quality of treatment >80%	76	44	9	50	67	44
Average quality of treatment ≥60% ≤80%	40	23	2	11	38	25
Poor quality of treatment <60%	55	32	7	39	48	31
**Areas of client satisfaction**						
Respectful behavior	113	66	13	72	100	65
Physical examination done	166	97	18	100	148	97
Satisfied with exam time	94	55	11	61	83	54
Satisfied with answers	155	91	16	89	139	91
Respectful behaviour of the facility staff	98	57	11	61	87	57
Client was satisfied	79	46	9	50	70	46

The standard protocol steps for counseling were at lowest level that extremely compromised the quality of the ANC services. Counseling for breast feeding was 6%, talked about contraception 6%, asked about place of delivery 31%, transport arrangement 20% and discussed about cost of delivery by 5% providers. TT was advised by 57% and next date of visits was advised by the 80% of the health care providers. The overall ranking of quality of counseling was poor in 92%, average in 6% and good in only 2% of the services provided.

Service delivery protocols practice for the treatment were relatively better than assessment and counseling but the large gaps were identified in treatment practices of the health care providers that includes Tetanus Toxoide (TT) vaccination, prescription for antimalarials, iron and folic acid tablets. Iron and folic acid and TT were prescribed by 69, 64 and 62% respectively but anti-malarial was advised by only by 2% of the health care providers in suspected malaria cases. The overall ranking of quality of treatment was poor in 32%, average in 23% and good in 44% of the services provided. Inferential statistical analysis showed that the overall gap to achieve above 80% level of counseling quality was 48 to 63% at 95% confidence limits. In response to questions in the client exit interview overall 46% clients perceived that they were satisfied with the provided ANC services. The proportion of clients who were satisfied with the services was almost similar in RHCs (50%) and BHUs (46%). (See detail in Table 2).

### Qualitative findings

The underlying factors for low coverage and quality explored from the clients, providers and managers. The translated views of each stakeholder are given in the Box-1. Distant location of facilities and lack of functional equipment, medicines and supplies was perceived by the clients, providers as well as health managers. Indifferent attitude and uncertainty in availability of the staff was complained by most of the clients. Community based indigenous ANC providers (Dais) are negatively influencing the ANC services enrollment due to affordability, availability 24/7 at door steps, providing massage, helping for household work and attending the deliveries at home. All these services can't be taken as substitute of the ANC services that needs examination from skilled persons and needs some lab diagnostic services from the health facility set-up. Lack of awareness and self decision power to visit the health facilities are also contributory factors for low coverage of ANC services in the rural area. Most of clients are visiting health facilities with expectation of being examined by ultrasound machine (USG) with hope to determine the sex of the fetus. The specific problems pointed out by providers were lack of guidance and supervision from the WMOs and facility in-charges and managers pointed out the deficiencies in district target setting, periodic monitoring systems, cumbersome purchase process and lack of training and skills.

## Discussion

Antenatal care is a very important component of maternal health services. It gives women and their families an opportunity to learn about the risks associated with pregnancy and guides their health seeking practices and decision making thereby preventing maternal and infant morbidity and mortality. Counseling during ANC provides a critical opportunity for women to learn when to seek help and where to give birth. It also helps prepare women for the mental and physical challenges they may face during pregnancy and childbirth. This study was focused on the current situation of coverage and quality of ANC services being provided to the rural mothers in the Punjab. ANC services are being offered by the government in rural areas of the Punjab through a network of PHC health facilities designated as ‘RHCs’ and ‘BHUs’. The private clinics/maternity homes and community health care providers are mainly providing delivery services because they are unable to provide the essential laboratory services package included in the standard ANC protocol. The rural women are mainly dependent on government's PHC health facilities for ANC services. The study finding revealed that overall 51.6% of the expected pregnancies first time reported for the ANC and out of those 33% didn't return back for follow-up. These findings straight way revealed that there is a gap of about 50% in the coverage of ANC services in the Punjab. The dropout in follow-up indicates the poor quality of the services that are annoying the clients not to return back for follow-up services. The overall coverage indicated in this study is very closer to that (57%) claimed by the HMIS cell Punjab [19] in DHIS report of 2010 and MICS 2007–2008, that is about (53%) [23]. This study was focused on rural areas and there was no opportunity for urban comparison but inequity in provision of ANC services to rural population have been reported in the literature [4]. The variation in coverage is associated with the number of standard ANC visits in assessment criteria e.g. in a study conducted in Alwar district of Rajasthan state, India, the practices of 3 or more ANC visits were lower in rural (36.1%) as compared to (71.4%) in urban areas [24].

Fifty percent ANC-1 registration of clients does not mean that the services are fulfilling quality of ANC services. It is just a registration figures. Although WHO recommends four ANC visits for every pregnant woman but the facility register contains only two columns one for the first visit and the other for revisits. Since all revisits are recorded in the same column, it is impossible to know how many of the registered cases completed four ANC visits. On the other hand, revisit/follow-up data indicates that about one third of the clients dropped out after the first visit, which means that a majority of the clients did not even complete two visits. Generally there were dropouts in revisits, in majority of the districts but in Rawalpindi there was an increase in revisits after the first visit. The reasons for this increase in revisits were the availability of Women Medical Officers, free facility of ultrasound, accessible locations, and availability of resources and awareness of the clients in these areas.

The ANC coverage was linearly increasing with better social developmental ranking a. composite proxy indicator [18]. The social factors have ‘push’ factor and quality of services have ‘pull’ factor for availing ANC services by the clients. Regarding quality of services the healthcare providers are not following the standard services delivery protocols during the history taking, clinical examination, treatment and counseling of the clients. Although about half of the clients were satisfied but that was their subjective perception that does not mean that quality services are being provided. Normative quality needs were not being met in more than 90% of the services being provided. The ultimate outcome of poor quality was reflected in the form of loss of follow-up for ANC. The loss of follow-up in about one 3rd of the enrolled clients raised a serious question on the quality of the services being provided. The evidence from another study supported that there was a significant association between satisfaction (outcome) and type of care, attitude of care provider, assessment of weight of women and waiting time [25]. The reasons identified for dissatisfaction among pregnant women in a public sector hospital of Hyderabad, Sindh were long waiting time, inadequate medicine supply and incomplete tetanus vaccination which were reported by 50% clients [19]. It is evident from a study conducted in Oman that short waiting time and positive behavior providers were the most satisfying aspects of the services [26]. Another study in India revealed that, the quality is a more significant predictor of utilization antenatal care services [17].

Reasons for low coverage at managerial level were distant location of health facilities, lack of functional equipment, medicine and supplies. Although government is providing services free or on nominal user charges, but the cost become unaffordable to marginralized clients due to unauthorized over changing for services in the PHC health facilities. Social factor effecting the coverage and drop-out at client level were lack of awareness and self decision power to visit the health facilities. The decisions about availing of ANC culturally endowed to the mother in law or husband. One of the factors responsible for the drop outs were indifferent attitude and uncertainty in availability of the staff.

Community based indigenous maternal care providers (Dais) are negatively influencing the ANC services enrolment due to affordability and availability 24/7 at door steps. The objective of ANC is to detect the high risk pregnancies and deliveries and if any services fail to do so can't be labeled as ANC whether provided at health facility or at home.

### Conclusion

The study revealed that both coverage and quality of the ANC services in Punjab are extremely inadequate. For coverage, only half of the expected pregnancies in the catchment areas of PHC health facilities are enrolled for ANC and out of those 1/3 never returned for follow-up visits. The quality of ANC is extremely compromised as perceived by clients and observed during the study. More than 50% of the clients are not satisfied with the ANC services they receive.

The major factors contributing for low coverage and quality at managerial and provider levels were distant location of health facilities, lack of functional equipment, insufficient supplies, inconvenient facility working hours and uncertainty in availability of staff. The social factors include lack of awareness, self decision power for seeking ANC, indifferent attitude of facility staff, affordability and availability of transport services. The miscellaneous home services provided by indigenous birth attendants are mistakenly considered as substitute of quality ANC, able to detect high risk pregnancies and deliveries.

Interventions are recommended to address the both coverage and quality issues depicted in the study findings. Health mangers can address the quality issues training, motivation and monitoring of health facility staff for client friendly behavior. Facility working hours must be extended and adjusted according to the convenience of clients in PHC health facilities. Removing transport barriers through sustainable public private partnerships or community development programs and retention of health care providers through incentivized package and facilities. Client awareness and self empowerment should be improved through involvement of local NGOs and social workers. For ANC client referral, the community based service health workers such as LHWs and CMWs must be targeted for advocacy and motivation for referring the clients to the public health facilities for ANC services.

To improve the quality of services health care providers should be trained to improve their technical skills for assessment, treatment and counseling of clients. Providers should get motivation and training to encourage them to follow the standard protocols for provision of quality ANC services.
